# Pooled Sample-Based GWAS: A Cost-Effective Alternative for Identifying Colorectal and Prostate Cancer Risk Variants in the Polish Population

**DOI:** 10.1371/journal.pone.0035307

**Published:** 2012-04-19

**Authors:** Pawel Gaj, Natalia Maryan, Ewa E. Hennig, Joanna K. Ledwon, Agnieszka Paziewska, Aneta Majewska, Jakub Karczmarski, Monika Nesteruk, Jan Wolski, Artur A. Antoniewicz, Krzysztof Przytulski, Andrzej Rutkowski, Alexander Teumer, Georg Homuth, Teresa Starzyńska, Jaroslaw Regula, Jerzy Ostrowski

**Affiliations:** 1 Department of Gastroenterology and Hepatology, Medical Center for Postgraduate Education, Warsaw, Poland; 2 Department of Oncological Genetics, Maria Sklodowska-Curie Memorial Cancer Center and Institute of Oncology, Warsaw, Poland; 3 Department of Urology, Maria Sklodowska-Curie Memorial Cancer Center and Institute of Oncology, Warsaw, Poland; 4 Department of Urology, Medical Center for Postgraduate Education, Warsaw, Poland; 5 Department of Colorectal Cancer, Maria Sklodowska-Curie Memorial Cancer Center and Institute of Oncology, Warsaw, Poland; 6 Interfaculty Institute for Genetics and Functional Genomics, University of Greifswald, Greifswald, Germany; 7 Department of Gastroenterology, Pomeranian Medical University, Szczecin, Poland; The Chinese University of Hong Kong, Hong Kong

## Abstract

**Background:**

Prostate cancer (PCa) and colorectal cancer (CRC) are the most commonly diagnosed cancers and cancer-related causes of death in Poland. To date, numerous single nucleotide polymorphisms (SNPs) associated with susceptibility to both cancer types have been identified, but their effect on disease risk may differ among populations.

**Methods:**

To identify new SNPs associated with PCa and CRC in the Polish population, a genome-wide association study (GWAS) was performed using DNA sample pools on Affymetrix Genome-Wide Human SNP 6.0 arrays. A total of 135 PCa patients and 270 healthy men (PCa sub-study) and 525 patients with adenoma (AD), 630 patients with CRC and 690 controls (AD/CRC sub-study) were included in the analysis. Allele frequency distributions were compared with t-tests and χ^2^-tests. Only those significantly associated SNPs with a proxy SNP (*p*<0.001; distance of 100 kb; r^2^>0.7) were selected. GWAS marker selection was conducted using PLINK. The study was replicated using extended cohorts of patients and controls. The association with previously reported PCa and CRC susceptibility variants was also examined. Individual patients were genotyped using TaqMan SNP Genotyping Assays.

**Results:**

The GWAS selected six and 24 new candidate SNPs associated with PCa and CRC susceptibility, respectively. In the replication study, 17 of these associations were confirmed as significant in additive model of inheritance. Seven of them remained significant after correction for multiple hypothesis testing. Additionally, 17 previously reported risk variants have been identified, five of which remained significant after correction.

**Conclusion:**

Pooled-DNA GWAS enabled the identification of new susceptibility loci for CRC in the Polish population. Previously reported CRC and PCa predisposition variants were also identified, validating the global nature of their associations. Further independent replication studies are required to confirm significance of the newly uncovered candidate susceptibility loci.

## Introduction

Cancers are highly heterogeneous, polygenic disorders that arise in a multi-step process involving the selection of successive cellular clones and result from genetic as well as specific environmental factors. In the former case, both high-penetrance mutations and low-penetrance polymorphisms may determine a patient's defense and adaptive mechanisms against exposure to carcinogenic factors, determining susceptibility to this disease. However, the effect of common low-penetrance risk determinants is small when in isolation, increasing susceptibility only through the cumulative effect associated with the occurrence of multiple risk variants [1].

The association between allele frequency and susceptibility to disease can be studied by focusing on individually selected variants or, instead, on the position of over a million DNA variants, using single nucleotide polymorphism (SNP) microarray technology. Microarray platforms used by genome-wide association studies (GWAS) represent a relatively mature technology that allows scanning the entire genome to detect potential associations with disease without prior knowledge of their position or biological function. In theory, as a consequence of linkage disequilibrium (LD) between SNPs at a given locus, a high proportion of all diversity could be captured by genotyping a relatively smaller subset of markers (the so-called tagging SNPs) [2]–[5].

To date, over 1,000 susceptibility loci, usually of small or modest effect and accuracy from low to moderately high, have been identified by GWAS [6]. However, each of these studies, including over 50 GWAS performed with cancer patients, identified only a few risk variants when analyzed separately. Moreover, many studies have not been replicated [7], [8]. The difficulties in the identification of genetic risk factors associated with heterogeneous and polygenic diseases, such as sporadic cancers, may be explained by the limitations of the methodology. Commercially available SNP array platforms have been optimized for studying diseases or traits based on the assumption that common diseases would be associated with common variants [9]. Since loci with a high effect size have been efficiently removed from the human population by natural selection, the identification of a common polymorphic susceptibility locus strongly associated with a disease, with odds ratio (OR) over 2 [10], is unlikely. Even though the identification of SNPs of low minor allele (MA) frequency have improved with the use of last generation chips, and higher probe densities enabled the study of variants with a low degree of heterozygosity, the detection of rare variants remains highly demanding in terms of statistical power [7], [8], [11]–[14].

Prostate cancer (PCa) and colorectal cancer (CRC) are the most common types of cancers in the Polish population, and the leading cause of cancer-related morbidity and mortality [15]. Most CRCs are sporadic, and only a small proportion occurs in the course of highly penetrating hereditary syndromes, such as Lynch syndrome, familial adenomatous polyposis and other polyposis syndromes mediated by rare germline mutations in the DNA mismatch repair gene and in the adenomatous polyposis coli (*APC*) gene [16]. PCa predisposition mediated through rare mutations in some candidate genes, such as the *BRCA2*, also explain less than 10% of the relative familial risk [17]. Therefore, it is possible that a substantial proportion of heritable cancer risk is explained by a combination of common low-penetrance variants of modest effects. For example, genetic variation in 14 and 21 independent susceptibility loci, validated in unrelated populations, may explain approximately 8% and 13.5% of the heritability risk of developing CRC and PCa, respectively [16], [18]. These results show, however, that most inherited variation associated with the risk of developing either cancer type remains to be determined.

A comprehensive analysis of variants conferring genetic susceptibility to CRC and PCa based on GWAS has not been conducted in the Polish population yet. A major cause for this lack of studies is the high cost of the SNP microarray technology, particularly considering that new loci identified by GWAS have been associated with progressively smaller effect sizes, demanding an increase in the statistical power (namely sample size) of GWAS. An alternative approach using pooled DNA samples has been developed [19]. Although the non-standard use of SNP arrays makes it necessary to take additional precautions into account [19], [20], this approach substantially reduces research costs. It is important to consider, however, that a higher technical variation associated with the DNA pooling approach may mask the weakest associations. Thus, researchers have to trade between accuracy of genetic risk prediction and cost of their research.

In this study, we describe a pooled DNA sample-based GWAS as a cost-effective alternative to identify genetic variants of moderate effect associated with CRC and PCa in the Polish population. Pooled DNA samples were processed using microarray technology, and GWAS was employed as a genetic variance filtering approach. The technical validation of the GWAS results and the replication studies on individual DNA samples was conducted using much cheaper PCR-based genotyping technology.

## Materials and Methods

### Ethics Statement

All enrolled patients and control subjects were Polish Caucasians recruited from two urban populations, Warsaw and Szczecin. The study was approved by the local ethics committee (Medical Center for Postgraduate Education and Cancer Center, Warsaw, Poland), and all participants provided written informed consent. The study protocol conforms to the ethical guidelines of the 1975 Declaration of Helsinki.

### Studied subjects

GWAS cohorts comprised: (1. AD/CRC sub-study) 525 patients (270 females and 255 males) diagnosed with colorectal adenomas (AD), 630 patients (240 females and 390 males) diagnosed with CRC and 705 healthy individuals (420 females and 285 males), and (2. PCa sub-study) 285 male patients diagnosed with PCa and 285 healthy men.

Larger cohorts of cases and controls were enrolled in a replication study, including: (1. AD/CRC sub-study) 945 (509 females and 436 males) patients with AD, 889 (352 females and 537 males) patients with CRC and 2188 (1542 females and 646 males) healthy individuals, and (2. PCa sub-study) 447 patients with PCa and 800 healthy men controls. The median age at diagnosis for AD, CRC and PCa was 60 years (range: 36–85), 64 years (range: 29–89) and 67 years (range: 42–83 years), respectively. Sample sizes and the age distribution of each group are shown in Table 1.

**Table 1:** Group statistics of the GWAS and the replication study cohorts.

	GWAS validation						Replication study					
	Enrolled			After TaqMan® filtration			Enrolled			After TaqMan® filtration		
	N	Range	Median	N	Range	Median	N	Range	Median	N	Range	Median
PCa	135	45–83	67	118	45–83	58	447	42–83	67	419	42–83	67
AD	525	27–85	59	476	27–85	59	945	32–85	60	856	36–85	60
AD (F)	270	28–85	58	242	28–85	58	509	32–85	60	454	40–85	60
AD (M)	255	27–85	60	234	27–85	60	436	36–85	61	402	36–85	61
CRC	630	29–86	65	598	29–86	65	889	28–89	64	840	29–89	64
CRC (F)	240	29–86	63	234	29–86	63	352	29–89	63	341	29–89	63
CRC (M)	390	32–84	66	364	32–84	66	537	28–85	65	499	30–85	65
Control - PCa	270	27–81	55	261	27–81	55	800	27–86	59	772	27–86	59
Control - AD/CRC	690	27–81	57	669	27–81	57	2188	21–87	58	1981	21–87	58
Control - AD/CRC (F)	420	40–77	58	408	40–77	58	1542	21–87	58	1399	21–87	58
Control - AD/CRC (M)	270	27–81	55	261	27–81	55	646	24–82	57.5	582	24–82	57

The GWAS validation panel indicates numbers of patients (N) enrolled in the GWAS, after excluding microarrays that did not meet quality control criteria based on the PCA results. The ‘Range’ and ‘Median’ values regard age of cases and controls in respective groups. Both GWAS validation and replication analyses were done using respective individual patient TaqMan® genotyping. The TaqMan® genotyping data was subjected to a quality filtration using the 5% threshold of per-individual maximum genotype missingness (see ‘*Statistical analyses – individual genotyping*’).

### Allelotyping GWAS

Genomic DNA was extracted from whole blood treated with EDTA using the QIAamp DNA Mini Kit (Qiagen, Germany), following the manufacturer's protocol. Before pooling, DNA sample concentrations were measured based on their fluorescent intensity using Quant-iT™ PicoGreen dsDNA Kit (Invitrogen, United Kingdom). To determine DNA quality with precision, the 260 nm/280 nm absorbance ratio of each sample was also measured using a NanoDrop 1000 spectrophotometer (Thermo Fisher Scientific Inc., USA), and samples were run on a 1% agarose gel to determine DNA integrity visually.

DNA samples that passed quality control tests were combined mixing equimolar concentrations according to patient diagnose to obtain 15-DNA sample pools. Pooled DNA samples were then brought to a final concentration of 50 ng/µl in Tris-EDTA buffer (pH = 8), with concentrations of Tris and EDTA not exceeding 10 mM and 0.1 mM, respectively. In the AD/CRC sub-study, a total of 35, 42 and 47 DNA pools were prepared for AD, CRC and controls, respectively, whereas in the PCa sub-study, a total of 19 and 19 DNA pools for both PCa and controls, respectively. To reduce the influence of experimental variation, DNA pools were subdivided into triple technical repeats and assayed independently, using separate microarrays, on the Affymetrix Genome-Wide Human SNP Array 6.0. Microarray genotyping experiments and the extraction of probe set signal intensities were performed using ATLAS Biolabs GmbH (Berlin, Germany).

### Individual genotyping

For the technical validation of GWAS findings and for the replication study, individual patients were genotyped using TaqMan SNP Genotyping Assays (Life Technologies, USA), SensiMix™ II Probe Kit (Bioline Ltd, United Kingdom), and a 7900HT Real-Time PCR system (Life Technologies, USA).

### Statistical analyses – allelotyping GWAS

The intensity of each SNP was calculated as the relative allele signal (RAS) for each microarray, such that: RAS = A/(A+B), where A and B are the probe set intensity values of alleles A and B, respectively, according to the Affymetrix coding [21], [22]. The intensity of A and B was obtained from the Affymetrix Birdseed v2 algorithm. Mean RAS values were next calculated for each DNA pool to account for the three technical repeats. Prior to conducting the association tests, a principal component analysis (PCA) for all arrays was performed based on RAS values. Pools identified as outliers by plotting the first two principal components were excluded from further analyses.

To detect significant differences in allele frequency between PCa and the control group a combination of two statistical approaches was used. Firstly, between-group differences in RAS were tested using Student's t-tests to take into account RAS variation among pools representing each group [23]. Secondly, mean RAS values of all arrays in the patient and control group were calculated and significant differences in allele frequency were tested using a χ^2^-test with one degree of freedom [24]. Since this test compares mean allele frequencies between groups without taking into account the high technical complexity of the allelotyping approach, it could lead to a higher number of false positive and false negative results. Conversely, the t-tests could be too sensitive to detect differences between groups if technical variation among pools is low. Thus, differences in allele frequency might be too small to be validated by individual genotyping. A combined statistical approach therefore provides a more accurate means to test for significant differences as compared to each test alone.

Candidate SNPs for individual genotyping were selected by combining the results from both the t-test and χ^2^-test, using the clumping algorithm in the PLINK v1.06 software (http://pngu.mgh.harvard.edu/purcell/plink) [25]. Those loci for which there was an SNP (*p*<0.001) and at least one correlated proxy SNP (r^2^>0.7) within a 100-kb region (*p*<0.001, χ^2^-test) were considered as positive results. Proxy SNPs were determined based on LD data obtained from 4100 individually genotyped Caucasian subjects from West-Pomerania in the SHIP cohort, using the Affymetrix Human SNP Array 6.0 [26], [27].

### Statistical analyses – individual genotyping

Technical validation of those candidate SNPs selected by the pooled-DNA GWAS was performed by individual genotyping of the same experimental cohorts. TaqMan genotyping data was first subjected to quality control procedures, including thresholds for maximum individual missingness for each of the SNPs <0.05, maximum genotype missingness for each of the individuals <0.05 and the Hardy-Weinberg disequilibrium <0.001 for the control group. GWAS candidate associations were validated using the allelic χ^2^-test (PLINK v1.07 software). SNPs with *p*-values <0.01 were eligible for further analyses. High levels of concordance in allele frequency differences between case and control groups validated the accuracy of the GWAS screening process, including the equimolar pool construction and the statistical approach for selection of candidate SNP associations.

Validated GWAS-derived SNPs and literature-selected SNPs (Table S1) were further analyzed by individual genotyping in the extended AD, CRC and PCa cohorts (Table 1). The binomial logistic regression model was used, using R software, to investigate associations in the context of additive gene action model for all the subjects enrolled in the study. A logistic regression analysis was also performed for PCa patients to determine whether any of the assayed SNPs was associated with early (<65 years of age) PCa onset. Benjamini-Hochberg correction was used for multiple comparisons.

The heterogeneity among study populations was assessed with the *I* ^2^ and *p*-value of the Cochran's Q statistic. For meta-analyses, pooled-OR values with 95% confidence intervals (CI) were calculated using *meta* function of STATA version 11. Their significance was assessed by Z test and *p*<0.05 was considered significant.

## Results

### Pooled-DNA allelotyping GWAS and individual DNA validation of the GWAS findings

The GWAS was carried out using pooled 15-DNA samples and the Affymetrix Genome-Wide Human SNP Array 6.0. The following outliers, identified by the PCA results, were excluded from the further analyses: 1) one pool representing 15 control male subjects in the AD/CRC sub-study and 2) 10 pools representing 150 PCa patients and one pool representing 15 controls, in the PCa sub-study. A reason why so many of PCa patient pools had to be rejected from further consideration is not clear. It can only be speculated that some pre-analytical variability, such as discreet changes in DNA quality and/or DNA microarray hybridization could affect the final results of the allelotyping experiments.

The pooled-DNA GWAS revealed 44 candidate SNPs associated with either AD, CRC or PCa, of which two were repeated in two unrelated comparisons. Considering SNP population frequencies of 0.2–0.5, our AD/CRC GWAS reached a power ranging from 98.6% to 99.8% and from 43% to 64% to detect effect size of OR = 2.0 and 1.5, respectively, at α = 1E-03, as estimated according to Dupont et al. [28] (Figure S1).

Next, the GWAS-selected SNPs were validated by genotyping of individual DNA samples using TaqMan SNP Genotyping Assays. Five candidate SNPs (rs2557030, rs2557227, rs2574608, rs2755895, rs7583683) were excluded from further statistical analysis due to significant deviations (*p*<0.001) from the Hardy-Weinberg equilibrium detected in the healthy control group. Although TaqMan genotyping-derived MA frequencies deviated slightly from the RAS values for MA obtained in the microarray experiment, there was an agreement in the direction of differences (OR) in the allele frequencies of the case and controls groups as shown by the allelic χ^2^-test (with *p*<0.01) for 30 out of 39 candidate SNPs: 24 associated with AD or CRC (one SNP, rs6702619, was identified in two separate comparisons) and six SNPs associated with PCa (Table 2).

**Table 2:** Pooled-DNA allelotyping GWAS and technical validation of GWAS selections using individual patient TaqMan genotyping.

G1 vs. G2	dbSNP ID^a^	Region	MA	Pooled-DNA GWAS					GWAS – technical validation			
				F1	F2	OR (95% CI)	*p*-value (t-test)	*p*-value (χ^2^)	F1	F2	OR (95% CI)	*p*-value (χ^2^)
PCa vs. N	rs1934636	1q32.2	C	0.464	0.356	1.57 (1.03–2.38)	1.31E-04	3.15E-03	0.373	0.233	1.96 (1.24–3.10)	4.50E-05
	rs12629904	3q13.31	T	0.196	0.13	1.63 (0.94–2.84)	1.25E-04	1.39E-02	0.079	0.03	2.77 (1.07–7.22)	2.58E-03
	rs1733329	3q13.33	T	0.347	0.284	1.34 (0.86–2.08)	1.41E-04	6.78E-02	0.332	0.23	1.66 (1.04–2.65)	2.37E-03
	rs1430579	4q31.21	C	0.408	0.279	1.78 (1.15–2.75)	2.42E-04	2.26E-04	0.341	0.211	1.93 (1.21–3.10)	9.10E-05
	rs667472	12p13.32	A	0.364	0.248	1.74 (1.11–2.71)	3.13E-04	5.37E-04	0.214	0.13	1.82 (1.05–3.17)	2.95E-03
	rs11616166	12p12.3	G	0.289	0.174	1.93 (1.19–3.14)	1.42E-05	1.53E-04	0.142	0.061	2.55 (1.25–5.17)	2.01E-04
AD vs. N	rs6762970	3p12.3	A	0.43	0.497	0.76 (0.61–0.96)	1.74E-05	9.29E-04	0.402	0.487	0.71 (0.56–0.90)	5.75E-05
AD vs. N (F)	rs7631421	3p14.1	C	0.361	0.452	0.68 (0.50–0.94)	2.26E-05	8.72E-04	0.259	0.344	0.67 (0.47–0.95)	1.49E-03
	rs2128834	3p22.1	G	0.183	0.242	0.7 (0.48–1.03)	2.77E-05	9.83E-03	0.116	0.206	0.51 (0.32–0.80)	3.31E-05
AD vs. N (M)	rs11876485	18q11.2	T	0.157	0.211	0.7 (0.45–1.09)	8.96E-06	2.52E-02	0.122	0.169	0.68 (0.41–1.14)	3.54E-02
	rs5975081	23q25	G	0.365	0.246	1.76 (1.21–2.57)	2.56E-04	2.89E-05	0.171	0.096	1.94 (1.14–3.31)	1.35E-02
CRC vs. N	rs6702619^b^	1p21.2	T	0.446	0.517	0.75 (0.61–0.93)	5.77E-07	2.39E-04	0.425	0.502	0.73 (0.59–0.92)	1.13E-04
	rs7611300^b^	3q26.33	A	0.28	0.223	1.36 (1.06–1.74)	1.63E-06	7.11E-04	0.018	0.013	1.39 (0.57–3.42)	3.32E-01
	rs13219695	6p21.2	G	0.321	0.387	0.75 (0.60–0.94)	8.14E-06	3.79E-04	0.109	0.169	0.6 (0.43–0.83)	1.59E-05
	rs2799652	6q16.1	A	0.499	0.434	1.3 (1.05–1.61)	2.35E-05	9.04E-04	0.338	0.276	1.34 (1.05–1.70)	6.89E-04
	rs879872	11p15.5	T	0.272	0.214	1.37 (1.07–1.77)	1.14E-11	4.44E-04	0.026	0.018	1.46 (0.86–3.12)	1.76E-01
	rs7171423	15q25.1	C	0.338	0.272	1.37 (1.08–1.73)	3.60E-05	2.27E-04	0.192	0.135	1.52 (1.13–2.06)	9.99E-05
	rs3803820	17q24.2	G	0.335	0.272	1.35 (1.07–1.71)	2.36E-04	4.24E-04	0.125	0.085	1.54 (1.07–2.21)	1.15E-03
	rs12689028	23p22.31	C	0.334	0.271	1.35 (1.07–1.71)	1.19E-08	4.88E-04	0.044	0.053	0.82 (0.49–1.38)	3.66E-01
	rs912956	23p11.1	C	0.437	0.374	1.3 (1.04–1.62)	1.93E-07	9.49E-04	0.247	0.2	1.31 (1.01–1.71)	1.47E-02
	rs5987543	23q22.2	C	0.411	0.348	1.31 (1.05–1.63)	8.02E-05	9.11E-04	0.161	0.127	1.32 (0.96–1.81)	4.05E-02
CRC vs. N (F)	rs9283670	4p13	C	0.346	0.26	1.51 (1.07–2.12)	1.41E-04	9.24E-04	0.109	0.065	1.76 (1.00–3.11)	5.85E-03
	rs17165506	7p21.3	G	0.293	0.202	1.64 (1.14–2.36)	3.41E-06	1.82E-04	0.154	0.081	2.07 (1.25–3.41)	5.54E-05
	rs441261	7p14.3	G	0.32	0.268	1.29 (0.91–1.82)	4.92E-06	4.47E-02	0.224	0.12	2.12 (1.38–3.25)	1.06E-06
CRC vs. N (M)	rs12994941	2p21	C	0.446	0.349	1.5 (1.09–2.07)	2.69E-05	4.17E-04	0.242	0.165	1.62 (1.08–2.42)	1.09E-03
	rs7611300^b^	3q26.33	A	0.283	0.201	1.57 (1.08–2.27)	1.74E-06	7.35E-04	0.019	0.016	1.19 (0.35–4.06)	6.23E-01
	rs40972	5q23.3	T	0.196	0.245	0.75 (0.52–1.09)	2.11E-05	3.09E-02	0.065	0.131	0.46 (0.27–0.80)	8.18E-05
	rs13192135	6p24.3	G	0.253	0.302	0.78 (0.55–1.11)	3.82E-06	5.08E-02	0.019	0.06	0.3 (0.12–0.75)	2.02E-04
	rs5978435	23p22.2	C	0.493	0.592	0.67 (0.49–0.92)	9.16E-06	3.88E-04	0.377	0.251	1.81 (1.27–2.57)	9.68E-04
CRC vs. AD	rs7533097	1p31.3	C	0.685	0.749	0.73 (0.51–1.03)	2.16E-04	6.91E-04	0.142	0.093	1.61 (1.10–2.37)	5.66E-04
	rs6702619^b^	1p21.2	T	0.446	0.528	0.72 (0.57–0.91)	3.29E-08	8.96E-05	0.425	0.533	0.65 (0.51–0.83)	6.62E-07
	rs9848984	3p26.3	C	0.676	0.754	0.68 (0.53–0.88)	1.19E-04	3.52E-05	0.077	0.04	2 (1.16–3.46)	3.89E-04
	rs11742611	5q11.2	G	0.516	0.58	0.77 (0.61–0.97)	1.09E-04	2.00E-03	0.416	0.514	0.67 (0.53–0.86)	7.10E-06
	rs10814948	9p24.2	T	0.702	0.634	1.36 (1.06–1.74)	1.87E-08	4.84E-04	0.252	0.346	0.64 (0.49–0.83)	2.47E-06
	rs1147451	14q23.3	T	0.681	0.621	1.3 (1.02–1.66)	5.12E-05	2.26E-03	0.287	0.368	0.69 (0.53–0.89)	6.72E-05
	rs5990890	23p22.12	G	0.672	0.732	0.75 (0.58–0.97)	1.58E-04	1.81E-03	0.126	0.086	1.53 (1.03–2.29)	1.23E-02
CRC vs. AD (F)	rs16860868	3q13.2	C	0.67	0.749	0.68 (0.48–0.96)	4.06E-05	5.67E-03	0.282	0.17	1.92 (1.24–2.98)	3.62E-05
CRC vs. AD (M)	rs6972867	7p12.2	C	0.543	0.623	0.72 (0.52–0.99)	5.19E-08	4.35E-03	0.239	0.153	1.74 (1.13–2.67)	3.55E-04
	rs7321756	13q31.2	G	0.271	0.185	1.64 (1.12–2.39)	1.59E-05	3.57E-04	0.865	0.929	0.49 (0.27–0.88)	4.73E-04

Technical validation was performed by individual typing of DNA samples from the same study cohorts used for pooled-DNA GWAS. The allele frequency distribution and χ^2^-test *p*-values were taken into account. G1 vs. G2; compared groups of cases and controls, respectively, MA; minor allele (+) strand, F1, F2; frequency of MA in the case and control groups, respectively, OR; odds ratio, CI; confidence interval, N; control, PCa; prostate cancer, AD; adenoma, CRC; colorectal cancer, F; female, M; male.

a ^/^SNP identifier based on NCBI SNP database;

b ^/^SNP identified in two independent comparisons.

### Replication study for GWAS-selected SNPs

Table 1 shows demographic details of subjects enrolled at the replication study. When a logistic regression was used to determine the significance of the association between the 30 GWAS-selected SNPs, using case or control as the dependent variable and appropriately coded TaqMan genotypes as independent variables, 17 SNPs were significantly (*p*<0.05) associated with AD or CRC in additive model of inheritance (Table 3). Seven of those SNPs remained significantly associated after multiple testing adjustment. The MA of three variants was associated with increased CRC susceptibility, whereas for four variants MA was associated with a decreased risk. When allele frequencies between cases and control subjects were assessed with the χ^2^-test corrected *p*-value, significant differences were observed for 13 SNPs (Table 3).

**Table 3:** The GWAS-selected SNPs association with AD, CRC or PCa, considering allelic and additive models.

							Allelic			Additive model			Meta-analysis
G1 vs. G2	dbSNP ID^a^	Region	Gene^b^	MA	F1	F2	OR (95% CI)	*p*-value	*p*-value_cor_.	OR (95% CI)	*p*-value	*p*-value_cor_.	*I^2^*(%) (Q *p*-value)
PCa vs. N	rs1934636	1q32.2	KCNH1 (*intron*)	C	0.290	0.245	1.26 (1.04–1.52)	**1.76E-02**	5.27E-02	1.14 (0.93–1.41)	2.09E-01	3.93E-01	81.2 (0.0212)
	rs12629904	3q13.31	*intergenic*	T	0.059	0.045	1.33 (0.92–1.94)	1.32E-01	2.83E-01	1.37 (0.92–2.05)	1.21E-01	3.03E-01	69.5 (0.0703)
	rs1733329	3q13.33	FSTL1	T	0.262	0.238	1.14 (0.94–1.38)	1.88E-01	3.14E-01	1.17 (0.94–1.44)	1.55E-01	3.32E-01	73.6 (0.0517)
	rs1430579	4q31.21	UCP1	C	0.264	0.242	1.12 (0.92–1.36)	2.45E-01	3.67E-01	1.07 (0.88–1.32)	4.92E-01	6.70E-01	87 (0.0055)
	rs667472	12p13.32	KCNA5	A	0.160	0.167	0.95 (0.75–1.19)	6.58E-01	7.59E-01	0.96 (0.75–1.24)	7.56E-01	8.76E-01	87 (0.0056)
	rs11616166	12p12.3	AEBP2 (*intron*)	G	0.080	0.069	1.18 (0.85–1.62)	3.23E-01	4.40E-01	1.05 (0.73–1.51)	7.93E-01	8.76E-01	84.3 (0.0116)
AD vs. N	rs6762970	3p12.3	CNTN3	A	0.418	0.450	0.88 (0.78–0.99)	**2.81E-02**	2.23E-01	0.85 (0.74–0.97)	**1.79E-02**	1.35E-01	76.8 (0.0379)
AD vs. N (F)	rs7631421	3p14.1	MITF	C	0.306	0.322	0.93 (0.79–1.10)	3.67E-01	8.03E-01	0.9 (0.75–1.10)	3.06E-01	8.91E-01	78.6 (0.0307)
	rs2128834	3p22.1	ULK4 (*intron*)	G	0.148	0.178	0.81 (0.65–0.99)	**4.07E-02**	3.56E-01	0.86 (0.68–1.09)	2.16E-01	8.91E-01	82.1 (0.0182)
CRC vs. N	rs6702619	1p21.2	PALMD	T	0.455	0.482	0.9 (0.80–1.01)	6.22E-02	2.36E-01	0.89 (0.78–1.01)	7.39E-02	2.10E-01	75.4 (0.0439)
	rs13219695	6p21.2	BTBD9 (*intron*)	G	0.117	0.153	0.73 (0.62–0.87)	**3.82E-04**	**7.26E-03**	0.71 (0.58–0.86)	**4.39E-04**	**1.49E-02**	42.6 (0.1869)
	rs2799652	6q16.1	FUT9	A	0.337	0.300	1.19 (1.05–1.34)	**6.04E-03**	**3.28E-02**	1.19 (1.03–1.36)	**1.49E-02**	1.01E-01	24.1 (0.2509)
	rs7171423	15q25.1	FAM108C1 (*intron*)	C	0.176	0.146	1.26 (1.08–1.47)	**3.88E-03**	**2.46E-02**	1.26 (1.06–1.50)	**8.39E-03**	9.51E-02	52.6 (0.1465)
	rs3803820	17q24.2	PRKCA (*intron*)	G	0.121	0.094	1.32 (1.10–1.58)	**2.87E-03**	**2.18E-02**	1.27 (1.03–1.56)	**2.24E-02**	1.27E-01	0 (0.3525)
CRC vs. N (F)	rs9283670	4p13	PHOX2B	C	0.094	0.081	1.17 (0.87–1.57)	2.89E-01	5.78E-01	1.16 (0.83–1.62)	3.99E-01	7.94E-01	60.5 (0.1117)
	rs17165506	7p21.3	TMEM106B	G	0.132	0.107	1.28 (0.99–1.64)	5.88E-02	2.35E-01	1.33 (1.00–1.76)	**4.68E-02**	1.87E-01	78.3 (0.0319)
	rs441261	7p14.3	SLC25A5	G	0.202	0.150	1.44 (1.16–1.78)	**8.70E-04**	**1.57E-02**	1.39 (1.09–1.78)	**8.18E-03**	9.88E-02	75.9 (0.0419)
CRC vs. N (M)	rs12994941	2p21	RPS12	C	0.233	0.214	1.12 (0.91–1.37)	2.95E-01	7.65E-01	0.98 (0.78–1.24)	8.72E-01	9.14E-01	76.3 (0.0402)
	rs40972	5q23.3	ADAMTS19 (*intron*)	T	0.072	0.123	0.55 (0.41–0.74)	**7.82E-05**	**2.81E-03**	0.55 (0.39–0.77)	**4.87E-04**	**1.56E-02**	0 (0.4839)
	rs13192135	6p24.3	BMP6 (*intron*)	G	0.021	0.040	0.52 (0.31–0.88)	**1.35E-02**	9.74E-02	0.47 (0.26–0.84)	**1.07E-02**	9.03E-02	31.4 (0.2272)
	rs5978435	23p22.2	ARHGAP6 (*intron*)	C	0.362	0.280	1.46 (1.13–1.89)	**4.25E-03**	5.10E-02	1.22 (1.05–1.41)	**1.13E-02**	9.03E-02	0 (0.3379)
CRC vs. AD	rs7533097	1p31.3	SGIP1	C	0.135	0.097	1.45 (1.17–1.80)	**6.30E-04**	**1.23E-02**	1.41 (1.10–1.80)	**6.85E-03**	**4.80E-02**	0 (0.5462)
	rs6702619	1p21.2	PALMD	T	0.455	0.507	0.81 (0.71–0.93)	**2.32E-03**	**2.03E-02**	0.79 (0.68–0.93)	**4.43E-03**	**3.87E-02**	75.2 (0.0446)
	rs9848984	3p26.3	CHL1	C	0.070	0.046	1.54 (1.15–2.07)	**3.64E-03**	**2.03E-02**	1.75 (1.24–2.48)	**1.63E-03**	**2.61E-02**	7 (0.2997)
	rs11742611	5q11.2	PELO	G	0.433	0.484	0.81 (0.71–0.93)	**2.89E-03**	**2.03E-02**	0.86 (0.73–1.00)	5.35E-02	2.08E-01	64.3 (0.0944)
	rs10814948	9p24.2	GLIS3	T	0.261	0.316	0.76 (0.66–0.89)	**4.49E-04**	**1.23E-02**	0.75 (0.63–0.90)	**1.54E-03**	**2.61E-02**	54.1 (0.1401)
	rs1147451	14q23.3	FUT8	T	0.294	0.346	0.79 (0.68–0.91)	**1.07E-03**	**1.39E-02**	0.8 (0.68–0.95)	**1.16E-02**	6.77E-02	13.6 (0.2819)
CRC vs. AD (F)	rs16860868	3q13.2	WDR52	C	0.257	0.195	1.42 (1.12–1.80)	**3.67E-03**	1.15E-01	1.31 (1.00–1.71)	**4.65E-02**	3.24E-01	55.4 (0.1345)
CRC vs. AD (M)	rs6972867	7p12.2	ZPBP	C	0.243	0.174	1.53 (1.21–1.93)	**3.38E-04**	**1.18E-02**	1.62 (1.22–2.14)	**8.36E-04**	**2.59E-02**	0 (0.5176)
	rs7321756	13q31.2	SLITRK5	G	0.131	0.101	1.35 (1.00–1.81)	**4.74E-02**	2.37E-01	1.28 (0.91–1.81)	1.51E-01	4.25E-01	63.4 (0.0986)

Bold denotes significant association (*p*<0.05). G1 vs. G2; compared groups of cases and controls, respectively, MA; minor allele (+) strand, F1, F2; frequency of MA in the case and control groups, respectively, OR; odds ratio, CI; confidence interval, N; control, PCa; prostate cancer, AD; adenoma, CRC; colorectal cancer, F; female, M; male.

a ^/^SNP identifier based on NCBI SNP database;

b ^/^NCBI ID of genes localized in proximity to the SNPs of interest (source: HapMap).

The statistical evidence for heterogeneity between allele frequencies across validation and replication study groups was assessed by the Q-test *p*-value. Of 30 GWAS-selected SNPs, 14 revealed overall low heterogeneity (*p*>0.1). Among them, significant associations in replication study cohorts were apparently more frequent, regardless the statistic used to determine the significance of association (Table 3). Lack of heterogeneity may be considered as a criterion of credible replication [29].

Six of the significantly associated SNPs were located within intronic gene regions: *BTBD9* (BTB/POZ domain-containing protein 9), *FAM108C1* (abhydrolase domain-containing protein), *PRKCA* (protein kinase C α; PKCα), *ADAMTS19* (a disintegrin and metalloproteinase with thrombospondin motif, member 19), *BMP6* (bone morphogenetic protein 6) and *ARHGAP6* (Rho GTPase-activating protein 6) (Table 3).

### Replication study of literature-selected SNPs

Thirty four and nine additional SNPs, previously shown to be associated with CRC [16], [30]–[45] and PCa [46]–[62] risk in various populations (Table S1), respectively, were also selected for the replication studies conducted using the same extended groups of cases and controls (Table 1). One SNP (rs6983267 at 8q24.21) was common for both tumor localizations. One SNP (rs10411210) was excluded from further analyses based on the result of the Hardy-Weinberg equilibrium test (*p*<0.001). Four other SNPs (rs36053993, rs2243250, rs2032582 and rs1057911) were also excluded from the logistic regression as they demonstrated at least a partial LD with other SNPs in the same region. They were therefore assigned with tagging SNPs, based on a SNP's lowest individual missingness ratio and the least significant Hardy-Weinberg test result for the control groups.

The association of 14 literature-selected variants with AD or CRC and four literature-selected variants with PCa was confirmed (*p*<0.05) in additive model of inheritance (Table 4). The association of the common SNP rs6983267 was confirmed for both the AD and PCa groups of patients. Strikingly, SNP rs1800894 (*IL10*) was associated in the opposite direction with AD and CRC susceptibility (Table 4). The MA of the remaining 10 variants was associated with an increased risk and six variants with a decreased risk of PCa, CRC and/or AD. Of these 17 variants, five (rs1800894, rs16892766, rs6983267, rs1859962 and rs4939827) remained significant after correction for multiple comparisons. When allele frequencies between cases and control subjects were assessed with the χ^2^-test corrected *p*-value, significant differences were observed in 11 comparisons for seven independent SNPs (Table 4).

**Table 4:** The literature-selected SNPs significant associations with AD, CRC or PCa, considering allelic and additive models.

					Allelic			Additive model		
dbSNP ID^a^	Region	Gene^b^	MA	G1 vs. G2	OR (95% CI)	*p*-value	*p*-value_cor_.	OR (95% CI)	*p*-value	*p*-value_cor_.
rs1800894	1q32.1	IL10 (*promoter*)	T	AD vs. N	0.67 (0.47–0.96)	2.77E-02	2.23E-01	0.58 (0.38–0.89)	1.24E-02	1.24E-01
				AD vs. N (F)	0.79 (0.5–1.25)	3.15E-01	8.03E-01	0.53 (0.30–0.94)	3.08E-02	3.19E-01
				CRC vs. N (F)	1.61 (1.09–2.39)	1.67E-02	9.42E-02	1.6 (1.05–2.44)	2.98E-02	1.48E-01
				CRC vs. AD	1.78 (1.21–2.64)	3.28E-03	**2.03E-02**	2.1 (1.30–3.37)	2.24E-03	2.61E-02
				CRC vs. AD (F)	2.04 (1.2–3.45)	7.01E-03	1.15E-01	3.03 (1.58–5.81)	8.51E-04	**2.55E-02**
rs373572	3p25.3	RAD18 (*exon*)	C	CRC vs. AD (M)	0.83 (0.67–1.02)	7.02E-02	2.62E-01	0.78 (0.61–0.99)	4.55E-02	2.44E-01
rs822395	3q27.3	ADIPOQ (*intron*)	C	CRC vs. N (F)	1.2 (1.01–1.43)	3.67E-02	1.65E-01	1.28 (1.05–1.55)	1.28E-02	1.03E-01
				CRC vs. AD (F)	1.3 (1.06–1.6)	1.29E-02	1.15E-01	1.33 (1.05–1.69)	1.70E-02	2.55E-01
rs2229992	5q21	APC (*exon*)	T	CRC vs AD (M)	1.26 (1.04–1.51)	1.81E-02	2.05E-01	1.26 (1.00–1.59)	4.75E-02	2.44E-01
rs16892766	8q23.3	EIF3H	C	CRC vs. N	1.63 (1.34–1.97)	6.27E-07	**2.38E-05**	1.45 (1.16–1.81)	9.71E-04	**1.65E-02**
				CRC vs. N (F)	1.76 (1.34–2.3)	4.11E-05	**1.48E-03**	1.53 (1.12–2.09)	7.13E-03	9.88E-02
				CRC vs. N (M)	1.5 (1.12–2.01)	7.08E-03	6.37E-02	1.43 (1.01–2.01)	4.26E-02	2.73E-01
				CRC vs. AD	1.34 (1.07–1.68)	1.09E-02	**4.73E-02**	1.39 (1.07–1.82)	1.48E-02	7.42E-02
rs6983267	8q24.21	*intergenic*	T	AD vs. N	0.84 (0.75–0.95)	3.39E-03	5.76E-02	0.84 (0.74–0.96)	1.14E-02	1.24E-01
				AD vs. N (F)	0.81 (0.69–0.94)	5.11E-03	8.94E-02	0.8 (0.68–0.95)	1.28E-02	1.98E-01
				PCa vs. N	0.77 (0.65–0.91)	2.07E-03	**1.04E-02**	0.75 (0.62–0.90)	2.49E-03	**1.87E-02**
rs1447295	8q24.21	*intergenic*	A	PCa vs. N	1.53 (1.18–1.97)	1.13E-03	8.49E-03	1.41 (1.06–1.86)	1.73E-02	6.49E-02
rs1057910	10q23.33	CYP2C9 (*exon*)	C	CRC vs. N (F)	1.51 (1.11–2.05)	8.12E-03	5.85E-02	1.51 (1.07–2.13)	1.97E-02	1.26E-01
				CRC vs. AD (F)	1.54 (1.05–2.25)	2.63E-02	1.49E-01	1.57 (1.02–2.41)	3.96E-02	3.24E-01
rs7931342	11q13.2	MYEOV	G	PCa vs. N	1.25 (1.05–1.47)	1.10E-02	**4.13E-02**	1.27 (1.05–1.53)	1.30E-02	6.48E-02
rs3802842	11q23.1	*intergenic*	C	CRC vs. AD	0.82 (0.7–0.95)	9.90E-03	**4.73E-02**	0.82 (0.69–0.97)	2.43E-02	1.06E-01
				CRC vs. AD (M)	0.79 (0.64–0.97)	2.56E-02	2.05E-01	0.77 (0.60–0.98)	3.30E-02	2.44E-01
rs7136702	12q13.13	LARP4	T	CRC vs. AD (M)	1.17 (0.96–1.44)	1.22E-01	3.57E-01	1.31 (1.03–1.67)	3.04E-02	2.44E-01
rs696	14q13.2	NFKBIA (*intron*)	T	CRC vs. N (F)	1.17 (0.98–1.38)	7.59E-02	2.73E-01	1.22 (1.02–1.47)	3.24E-02	1.48E-01
rs4779584	15q13.3	*intergenic*	T	AD vs. N (M)	1.24 (1.01–1.54)	4.28E-02	3.53E-01	1.34 (1.05–1.70)	1.86E-02	5.04E-01
				CRC vs. N (M)	1.34 (1.1–1.63)	3.66E-03	5.10E-02	1.37 (1.09–1.73)	7.46E-03	9.03E-02
rs9929218	16q22.1	CDH1 (*intron*)	A	AD vs. N	0.88 (0.78–1)	5.05E-02	2.23E-01	0.86 (0.75–1.00)	4.39E-02	2.63E-01
				AD vs. N (M)	0.84 (0.69–1.02)	8.08E-02	5.33E-01	0.77 (0.60–0.98)	3.48E-02	5.04E-01
rs1859962	17q24.3	*intergenic*	T	PCa vs. N	0.73 (0.62–0.87)	4.20E-04	**6.30E-03**	0.73 (0.61–0.89)	1.57E-03	**1.87E-02**
rs4939827	18q21.1	SMAD7 (*intron*)	C	AD vs. N	0.81 (0.72–0.9)	1.98E-04	**6.71E-03**	0.82 (0.72–0.94)	3.86E-03	1.16E-01
				AD vs. N (F)	0.72 (0.62–0.84)	1.96E-05	**6.85E-04**	0.76 (0.64–0.90)	1.54E-03	**4.78E-02**
				CRC vs. N	0.83 (0.74–0.94)	1.87E-03	**2.18E-02**	0.85 (0.75–0.96)	1.20E-02	1.01E-01
				CRC vs. N (F)	0.79 (0.67–0.94)	7.19E-03	5.85E-02	0.78 (0.65–0.94)	9.26E-03	9.88E-02
rs961253	20p12.3	*intergenic*	A	CRC vs. AD (M)	1.2 (0.98–1.46)	7.50E-02	2.62E-01	1.33 (1.05–1.68)	1.65E-02	2.44E-01

Bold denotes significant association (*p*-value_cor_<0.05). MA; minor allele (+) strand, G1 vs. G2; compared groups of cases and controls, respectively, OR; odds ratio, CI; confidence interval, N; control, PCa; prostate cancer, AD; adenoma, CRC; colorectal cancer, F; female, M; male.

a ^/^SNP identifier based on NCBI SNP database;

b ^/^NCBI ID of genes localized in proximity to the SNPs of interest (source: HapMap).

To validate the global nature of these associations, between-dataset heterogeneity was tested. In the meta-analysis we included three SNPs associated with CRC and four SNPs associated with PCa susceptibility in our replication study for which associations were found with the same phenotype in at least four other studies. A random-effects model was used to calculate the pooled-OR values. As shown in Table 5, lack of demonstrable heterogeneity (Q *p*-value of less than 0.1) was noted across datasets representing three out of seven SNPs, and all pooled-ORs were significant (*p*<0.001).

**Table 5:** Meta-analysis of previously reported PCa and CRC associations including replication results from the present study.

			Random effects		Heterogeneity			
dbSNP ID^a^	Risk allele	Phenotype	OR (95% CI)	Z*p*-value	Q*p*-value	*I* ^2^ (%)	No. of studies	References
rs1447295	A	PCa vs. N	1.45 (1.33–1.57)	<0.001	0.139	9.676	7	[47], [52], [57]–[60]
rs6983267	G	PCa vs. N	1.26 (1.19–1.33)	<0.001	0.013	19.373	9	[47], [52], [53], [57], [59], [61], [62]
rs7931342	G	PCa vs. N	1.19 (1.14–1.24)	<0.001	0.676	3.157	6	[49], [55], [56], [61]
rs1859962	G	PCa vs. N	1.24 (1.17–1.31)	<0.001	0.313	4.757	5	[47], [49], [52], [57]
rs16892766	C	CRC vs. N	1.27 (1.23–1.32)	<0.001	0.691	3.059	6	[16], [36], [41], [42], [45]
rs4779584	T	CRC vs. N	1.20 (1.53–1.25)	<0.001	0.092	13.61	9	[16], [35], [36], [41]–[43]
rs4939827	C^b^	CRC vs. N	0.84 (0.81–0.88)	<0.001	0.015	18.95	9	[16], [32], [33], [36], [42], [44]

a ^/^SNP identifier based on NCBI SNP database;

b ^/^meta-analysis was done for minor allele (MA).

To check whether any of the studied variants was associated with an early age of PCa onset, we performed a logistic regression analysis including cases only, with a binary indicator for age (below or above 65 years of age, coded as 1 and 0, respectively) at PCa diagnosis and the studied SNPs as independent variables. There were 171 patients diagnosed at age 65 or earlier and 247 patients older than 65. Two SNPs were significantly associated with age at PCa diagnosis (Table S2): rs1934636 and rs6983267. The former, a GWAS-selected SNP, was more frequent in the group of older patients (OR = 0.6, 95% CI 0.39–0.93, *p* = 2.18E-02), considering the dominant gene action model. Conversely, the rs6983267 variant was associated with a younger patient age in the age-stratified analysis; OR = 1.40, 95% CI 1.01–1.95, *p* = 4.44E-02).

## Discussion

### Pooled DNA-based GWAS utility

It is generally accepted that well-designed GWAS should be conducted with groups of at least 1,000 patients and 1,000 controls, even though appropriate levels of statistical power to test for genetic associations (at *p*<5E-08) often relate to higher effect sizes [14]. These GWAS significance thresholds result from the requirement to correct for multiple comparisons and are aimed at minimizing the number of false positive findings [8]. However, exceedingly restrictive statistical criteria may, in turn, produce false negative results [11]–[13]. Indeed, those significant associations from independent replication studies were not ranked in the top 1,000 SNPs in the initial GWAS [46]. Thus, the use of stringent criteria may prevent the detection of subtle associations and account for missing heritability [14]. It is also recognized that there is certain level of heterogeneity in the GWAS results, which may arise due to the different genetic background (population stratification) of geographically distinct populations [41], [63], [64], or because of the bias introduced by population admixture effects [65], [66]. Although few CRC susceptibility loci (as 8q24.21, 8q23.3 or 18q21.1) have been replicated in a number of studies [41], it is symptomatic that some of the identified associations reflect between-populations differences in tumor sub-site, age of CRC/AD onset, sex or smoking status within the groups studied [41]. Thus, large cohort studies can ignore some sub-population-specific risk variants, so genome-wide genotyping should be also conducted in smaller cohorts. Conversely, studies with lower sample sizes typically reveal a smaller fraction of the heritability of a complex disease by failing to detect associations that do not reach statistical significance [7].

Since the final GWAS results depend on many factors, each associated with a different stage of the experimental procedure, their analysis and interpretation are often challenging. It is essential to realize that the GWAS results reflect, at best, the differences in the genetic material of the cases and controls used for analysis. Although this may seem obvious, it emphasizes one of the most fundamental conditions required for a successful GWAS. Therefore, precise diagnostic criteria must be employed to obtain homogenous groups, as a nonrandom distribution of individuals with traits governed by strong genetic determinants, such as single-gene mutations, will strongly bias the final GWAS outcome.

Although our pooled DNA-based GWAS represent studies with small sample size, they identified 30 SNPs significantly overrepresented in the studied groups (Table 2), which were further validated by TaqMan genotyping of the individual DNA samples. The replication studies selected 17 candidate risk variants associated with CRC, considering additive model of inheritance (Table 3). These associations had not been previously reported. Seven of them remained significant after correction for multiple hypothesis testing.

Although not all GWAS-selected susceptibility SNPs will have a direct functional association with a cancer phenotype, a careful analysis of the GWAS results showed that those SNPs located in intronic regions or in the LD blocks with nearby genes have a potential to influence cancer development (Table 3). Noteworthy, several candidate susceptibility genes (*PRKCA*, *BMP6*, *ADAMTS19*, *ARHGAP6*, *FUT9/8*, *FAM108C1*, *CHL1*, *BTBD9* and *WDR52*) are involved in the actin cytoskeleton arrangement, cell adhesion and cell motility processes, which are important for cancer invasion and metastasis.

The rs3803820 located in the *PRKCA* gene (17q24.2) was selected in the CRC sub-study, showing OR = 1.27 (*p* = 2.24E-02). Other candidate SNP rs13192135, which showed a strong effect size of OR = 0.47 (*p* = 1.07E-02) in the CRC male group, is located at 6p24.3 in the intronic region of the *BMP6* gene. Similarly, strong association with both AD and CRC risk, of the known rs4939827 variant of *SMAD7* gene was indicated in the present study (Table 4). This is in agreement with several previous studies showing association of genetic variation in the BMP/Smad pathway-related genes with CRC risk [32], [33], [67].

The rs9848984 SNP at 3p26.3, downstream to the close homolog of L1 (*CHL1*) gene, is located in the LD block involving the 3′-end of the gene. CHL1 is involved in cancer growth and in the metastasis of different human cancers, including colon and breast cancers [68]. The observation that both mRNA and protein levels of ARHGAP6 were elevated in the CRC tissue and cell lines suggests that it may serve as a biomarker for the development and progression of CRC [69]. Similarly, a high level of metalloprotease *ADAMTS19* expression was observed in several tumor tissues and cell lines [70]. In turn, FAM108C1 activity was shown to predict the development of distant metastases [71].

The rs2799652 SNP was found in the promoter region of the alpha-(1,3)-fucosyltransferase (*FUT9*) gene, responsible for the biosynthesis of the Lewis X antigen, a cancer-associated antigen expressed preferentially in premalignant colon polyps [72]. FUT8, in turn, is responsible for modulation of E-cadherin function [73]. Previous studies showed that *FUT8* and E-cadherin expression levels were significantly higher in primary CRC samples and that E-cadherin core fucosylation enhanced cell-cell adhesion in colon carcinoma [74]. Both *FUT9* and downstream to *FUT8* gene variations were shown to be associated with CRC risk in this study (Table 3). Interestingly, our replication study revealed also association between the intronic sequence variation (rs9929218) in the E-cadherin gene (*CDH1*) and AD risk, especially in males (Table 4).

We replicated previously reported associations between four PCa and 14 AD/CRC risk variants in our Polish-based cohorts. Four SNPs (rs1859962, rs7931342, rs1447295 and rs6983267) were widely reported as PCa risk variants in Caucasian, African or Asian populations [46], [48]–[51], [55]–[58], and can be considered global markers of PCa susceptibility. In the case of CRC, 11 susceptibility loci were reported often in previous studies [41]. Seven of these loci were replicated in the present study: 8q23.3, 8q24.21, 11q23.1, 15q13.3, 16q22.1, 18q21.1, 20p12.3. In a Swedish-based cohort study, five of the same 11 loci showed a significant OR [42]. The lack of confirmation of loci 11q23.1, 16q22.1 and 20p12.3 in the Swedish study may have resulted from their association with cancer risk mostly in men, unlike in woman, and/or because they are associated with AD rather than CRC risk, as indicated by our findings (Table 4).

Interestingly, the stratified analyses revealed that the rs4939827 (18q21.1) variant's association was limited to women only (OR = 0.6, 95% CI 0.42–0.88, *p* = 0.007) [75], indicating that common genetic variants in *SMAD7* may confer susceptibility to colon cancer particularly among women. In another study, rs9929218 at 16q22.1 (*CDH1*) was most strongly associated with risk in male than in female subjects [41]. Similarly in this study, rs4939827 was associated both with AD risk (OR = 0.76, *p* = 1.54E-03) and CRC risk (OR = 0.78, *p* = 9.26E-03) among female patients, whereas rs9929218 was associated with AD risk in men (OR = 0.77, *p* = 3.48E-02) (Table 4). Additionally, among females only at least two significant association were observed for rs1800894 (1q32, *IL10*), rs822395 (3q27.3, *ADIPOQ*) and rs1057910 (10q23.33, *CYP2C9*). Conversely, among males, at least two associates were shown for the rs4779584 (15q13.3) variant. Our results support the notion that specific variants serve as gender-specific markers predisposing to CRC.

SNPs rs1447295 and rs6983267 are located at the 8q24 region. Several studies have identified 8q24 as an important region associated with risk for various cancers, including prostate, breast, colon, ovarian and bladder cancers [62], [76]–[78]. To date, all susceptibility markers within 8q24 were located at five distinct LD blocks [53]. SNP rs1447295 is located at block 5 (previously referred as susceptibility region 1) and was shown to increase PCa risk in various populations with an OR ranging from 1.21 to 1.81, [47], [48], [57]–[60]. Its rare allele A was also shown to be associated with an increased risk for prostate-specific antigen (PSA) recurrence in patients receiving radical prostatectomy (OR = 1.56, 95% CI 1.14–2.21) [79]. In fact, a meta-analysis of this SNP supported previously GWAS-reported associations [80].

Among the polymorphisms in block 4 (region 3) at 8q24, rs6983267 has been consistently identified in many studies, with an OR ranging from 0.65 to 1.42 [46], [47], [49]–[51], [57], [59], [81], [82], therefore the strongest association with PCa risk in this LD block [53], [83]. It has also been associated with CRC and ovarian cancer [76]. Recently, a meta-analysis showed an allelic and genotypic association of the rs6983267 polymorphism with CRC risk among Asians, Europeans, and Americans with a European ancestry [82]. Surprisingly, this variant did not show any association with the CRC phenotype in our study. However, it was significantly associated with AD risk (in the whole group and among females only) (Table 4). In our age-stratified analysis, the minor allele T of rs6983267 was significantly associated with a younger age at PCa diagnosis (≤65 years; considering an additive mode of inheritance) (Table S2). Accordingly, the G allele of rs6983267 was associated with an older age at PCa diagnosis in the Swedish population [42], and the higher PCa risk associated with this SNP was approximately doubled in those individuals susceptible to an early disease onset or to the development of a clinically aggressive disease [84].

Only a few studies examine the association between rs1447295 and PCa risk and between rs6983267 and both PCa and CRC risk in the Polish population [85]–[87]. In line with our results, significant associations were observed for allele A of rs1447295 (OR = 1.3, 95% CI 1.1–1.6, *p* = 0.01) [85], [86], and between allele G of rs6983267 and PCa (OR = 1.43, 95% CI 1.23–1.66, *p* = 10^−9^) and CRC (OR = 1.13, 95% CI 0.93–1.37, *p* = 0.01) risk [85], [87].

Still, some previously reported associations with CRC and PCa risk were not replicated in our study. This may have been a result of a low statistical power coupled with a high genetic heterogeneity and/or cancer complexity [8]. If so, these inconsistencies may stem from a potential hidden stratification of our cohort, despite the apparent homogeneity of the Polish population.

### Utility of cancer risk variants revealed by GWAS

The only factor that decreases cancer-related mortality significantly is early diagnosis. Since at the early stage of development cancers are asymptomatic or associated with unspecific symptoms, early diagnosis is usually accidental or results from the participation in screening programs. Epidemiological studies demonstrate that screening can be effective in a few cancer locations, including the large bowel and prostate. However, screening effectiveness depends not only on the availability of appropriate diagnostic tests, but also on the general acceptance of the proposed screening methods by those who consider themselves healthy. Colonoscopy used for CRC screening also allows simultaneous detection and removal of ADs, but it is a rather expensive procedure with low acceptability, especially by men [88]. By contrast, simple and cheap detection of serum PSA is widely accepted as a screening tool, but its predictive value is limited by the lack of specificity and the inability to differentiate indolent from aggressive PCa [89]. Therefore, specific but more expensive imaging-based methods might be introduced in PCa preventive programs. Enrolling healthy individuals with a higher risk of cancer to screening programs would increase the acceptance of screening exams, and therefore enhance their effectiveness and greatly reduce healthcare costs. Currently, CRC screening guidelines are based on age and to some extent on the family history of screeners. These guidelines could be also customized according to gender, race, ethnicity, smoking habits and presence of obesity, diabetes and metabolic syndromes [90].

One of the early hopes of the GWAS approach was to enable the development of risk prediction models that could accurately select high-risk individuals based on their genetic profiles. However, the proportion of risk explained by known susceptibility variants is still small. For example, according to a recently published meta-analysis of 30 selected SNPs associated with PCa risk, the proportion of the total genetic variance attributed to each SNP ranged from 0.2% to 0.9% as based on both OR and risk allele frequency [18]. Moreover, since the relative risk conferred by these loci is moderate or low, with ORs below 2, and new loci identified by GWAS have had progressively smaller effect sizes, the capacity for risk prediction in newly discovered common marker SNPs may be diminishing [89]. The problem is further complicated by interactions between genetic and environmental risk factors, largely due to a lack of established guidelines or procedures that would determine the impact of environmental factors on humans over the span of a lifetime. Thus, the information provided by genome-wide genotyping is often insufficient to be clinically useful in the prediction of cancer. In this sense, the cost of GWAS-based studies should be always considered, especially when adequate GWAS coverage of risk variants of small or modest effect requires larger sample sizes.

The major idea behind genomic studies is not only to enable recognizing genetic variability associated with susceptibility to a disease, but also to recognize the complex nature of genetic variability underlying its pathogenesis [1]. In this regard, although the genetic variants identified to date explain only a modest proportion of cancer heritability, their combination with additional, newly discovered loci may have a greater, cumulative, effect. Ideally, instead of typing all known variants, the most informative combination of potential SNPs should be assessed. Further research is therefore needed to enable the detection of new susceptibility variants. Moreover, it would be beneficial if such efforts were accompanied by an increase in the statistical power of GWAS.

In summary, in this study we provide evidence for the utility of pooled sample-based GWAS instead of genome-wide genotyping of individual DNA samples as a cost-effective alternative approach for filtering genetic variance which reached a decent statistical power particularly for the relatively common SNP markers of moderate effect sizes. The usefulness of pooling-based GWAS was exemplified through the identification of SNPs associated with CRC and PCa susceptibility in the Polish population. However, considering the complex nature of cancer, which involves the interaction of different genetic and environmental factors, detecting all cancer markers present in the human genome is a task beyond capabilities. In addition to previous findings, the risk information provided in the present study is still not sufficient to be used in clinical practice.

## Supporting Information

Table S1 Literature-selected SNPs used in the replication study.
(DOC)

Table S2 SNP association with early PCa onset (before 65 years of age) considering additive (ADD), dominant (DOM), or recessive (REC) models of gene action.
(DOC)

Figure S1 Statistical power of the AD/CRC GWAS for alleles found at different frequencies in the general population (p0).
(TIF)
