# Supplementary material for: Pooled Sample-Based GWAS: A Cost-Effective Alternative for Identifying Colorectal and Prostate Cancer Risk Variants in the Polish Population
Source: PLoS One. 2012 Apr 19;7(4):e35307. doi: 10.1371/journal.pone.0035307 (PMC3331859; doi:10.1371/journal.pone.0035307)
Supplement: Table S1 — Literature-selected SNPs used in the replication study. (DOC) [file pone.0035307.s001.doc]

| **AD/CRC** | | **PCa** | |
| --- | --- | --- | --- |
| **dbSNP ID** | **Region** | **dbSNP ID** | **Region** |
| rs34612342 | 1p34.1 | rs10486567 | 7p15.2 |
| rs36053993 | 1p34.1 | rs6983561 | 8q24.21 |
| rs1342387 | 1q32.1 | rs1447295 | 8q24.21 |
| rs1800894 | 1q32.1 | rs6983267 | 8q24.21 |
| rs1800872 | 1q32.1 | rs10993994 | 10q11.23 |
| rs6691170 | 1q41 | rs7931342 | 11q13.2 |
| rs6687758 | 1q41 | rs4430796 | 17q12 |
| rs373572 | 3p25.3 | rs1859962 | 17q24.3 |
| rs10936599 | 3q26.2 | rs5945619 | 23p11.22 |
| rs822395 | 3q27.3 |  |  |
| rs2229992 | 5q21 |  |  |
| rs2070874 | 5q31.1 |  |  |
| rs2243250 | 5q31.1 |  |  |
| rs1128503 | 7q21.12 |  |  |
| rs2032582 | 7q21.12 |  |  |
| rs16892766 | 8q23.3 |  |  |
| rs6983267 | 8q24.21 |  |  |
| rs719725 | 9p24.1 |  |  |
| rs10795668 | 10p14 |  |  |
| rs1057910 | 10q23.33 |  |  |
| rs1057911 | 10q23.33 |  |  |
| rs7903146 | 10q25.2 |  |  |
| rs3802842 | 11q23.1 |  |  |
| rs7136702 | 12q13.13 |  |  |
| rs11169552 | 12q13.13 |  |  |
| rs696 | 14q13.2 |  |  |
| rs4444235 | 14q22.2 |  |  |
| rs4779584 | 15q13.3 |  |  |
| rs9929218 | 16q22.1 |  |  |
| rs13894 | 17p13.1 |  |  |
| rs4939827 | 18q21.1 |  |  |
| rs10411210 | 19q13.11 |  |  |
| rs961253 | 20p12.3 |  |  |
| rs4925386 | 20q13.33 |  |  |
