# Supplementary material for: Pooled Sample-Based GWAS: A Cost-Effective Alternative for Identifying Colorectal and Prostate Cancer Risk Variants in the Polish Population
Source: PLoS One. 2012 Apr 19;7(4):e35307. doi: 10.1371/journal.pone.0035307 (PMC3331859; doi:10.1371/journal.pone.0035307)
Supplement: Table S2 — SNP association with early PCa onset (before 65 years of age) considering additive (ADD), dominant (DOM), or recessive (REC) models of gene action. (DOC) [file pone.0035307.s002.doc]

| **dbSNP ID^a^** | **Region** | **MA** | **Model** | **OR** | **95% CI** | ***p*-value** |
| --- | --- | --- | --- | --- | --- | --- |
| **rs1934636** | **1q32.2** | C | ADD | 0.76 | 0.54-1.07 | 1.14E-01 |
|  |  |  | **DOM** | **0.60** | **0.39-0.93** | **2.18E-02*** |
|  |  |  | REC | 0.80 | 0.36-1.74 | 5.68E-01 |
| rs12629904 | 3q13.31 | T | ADD | 0.84 | 0.43-1.62 | 6.02E-01 |
|  |  |  | DOM | 0.81 | 0.40-1.64 | 5.53E-01 |
|  |  |  | REC | nd | nd | nd |
| rs1733329 | 3q13.33 | T | ADD | 0.98 | 0.69-1.40 | 9.05E-01 |
|  |  |  | DOM | 1.02 | 0.66-1.58 | 9.18E-01 |
|  |  |  | REC | 0.80 | 0.31-2.06 | 6.42E-01 |
| rs1430579 | 4q31.21 | C | ADD | 1.04 | 0.73-1.48 | 8.26E-01 |
|  |  |  | DOM | 1.03 | 0.66-1.61 | 9.09E-01 |
|  |  |  | REC | 1.31 | 0.58-2.93 | 5.13E-01 |
| rs10486567 | 7p15.2 | A | ADD | 1.06 | 0.72-1.55 | 7.69E-01 |
|  |  |  | DOM | 1.03 | 0.65-1.63 | 8.88E-01 |
|  |  |  | REC | 1.23 | 0.43-3.48 | 6.97E-01 |
| rs6983561 | 8q24.21 | C | ADD | 1.46 | 0.62-3.44 | 3.87E-01 |
|  |  |  | DOM | 1.33 | 0.56-3.13 | 5.18E-01 |
|  |  |  | REC | nd | nd | nd |
| rs1447295 | 8q24.21 | A | ADD | 1.00 | 0.64-1.57 | 1.00E+00 |
|  |  |  | DOM | 0.95 | 0.57-1.59 | 8.47E-01 |
|  |  |  | REC | 1.61 | 0.41-6.37 | 4.98E-01 |
| **rs6983267** | **8q24.21** | T | **ADD** | **1.44** | **1.04-1.99** | **2.65E-02*** |
|  |  |  | **DOM** | **1.83** | **1.11-3.02** | **1.87E-02*** |
|  |  |  | REC | 1.39 | 0.81-2.41 | 2.34E-01 |
| rs10993994 | 10q11.23 | T | ADD | 0.83 | 0.60-1.15 | 2.59E-01 |
|  |  |  | DOM | 0.98 | 0.63-1.55 | 9.45E-01 |
|  |  |  | REC | 0.55 | 0.27-1.10 | 8.89E-02 |
| rs7931342 | 11q13.2 | G | ADD | 0.98 | 0.71-1.35 | 8.94E-01 |
|  |  |  | DOM | 1.08 | 0.64-1.83 | 7.61E-01 |
|  |  |  | REC | 0.86 | 0.52-1.43 | 5.72E-01 |
| rs667472 | 12p13.32 | A | ADD | 1.43 | 0.93-2.19 | 1.07E-01 |
|  |  |  | DOM | 1.42 | 0.88-2.28 | 1.50E-01 |
|  |  |  | REC | 2.74 | 0.62-12.20 | 1.85E-01 |
| rs11616166 | 12p12.3 | G | ADD | 0.98 | 0.53-1.81 | 9.46E-01 |
|  |  |  | DOM | 0.92 | 0.49-1.72 | 7.87E-01 |
|  |  |  | REC | nd | nd | nd |
| rs4430796 | 17q12 | G | ADD | 0.96 | 0.70-1.32 | 8.07E-01 |
|  |  |  | DOM | 0.80 | 0.51-1.25 | 3.26E-01 |
|  |  |  | REC | 1.19 | 0.67-2.12 | 5.42E-01 |
| rs1859962 | 17q24.3 | T | ADD | 0.74 | 0.53-1.02 | 6.78E-02 |
|  |  |  | DOM | 0.70 | 0.45-1.09 | 1.13E-01 |
|  |  |  | REC | 0.70 | 0.35-1.37 | 2.92E-01 |
| rs5945619 | Xp11.22 | C | ADD | 1.18 | 0.75-1.86 | 4.72E-01 |
|  |  |  | DOM | nd | nd | nd |
|  |  |  | REC | nd | nd | nd |

*Bold denotes significant association (*p* < 0.05). MA; minor allele (+) strand, OR; odds ratio, CI; confidence interval, nd; not detected.

^a/^ SNP identifier based on NCBI SNP database.
