# Supplementary figures and images for: Pooled Sample-Based GWAS: A Cost-Effective Alternative for Identifying Colorectal and Prostate Cancer Risk Variants in the Polish Population
Source: PLoS One. 2012 Apr 19;7(4):e35307. doi: 10.1371/journal.pone.0035307 (PMC3331859; doi:10.1371/journal.pone.0035307)

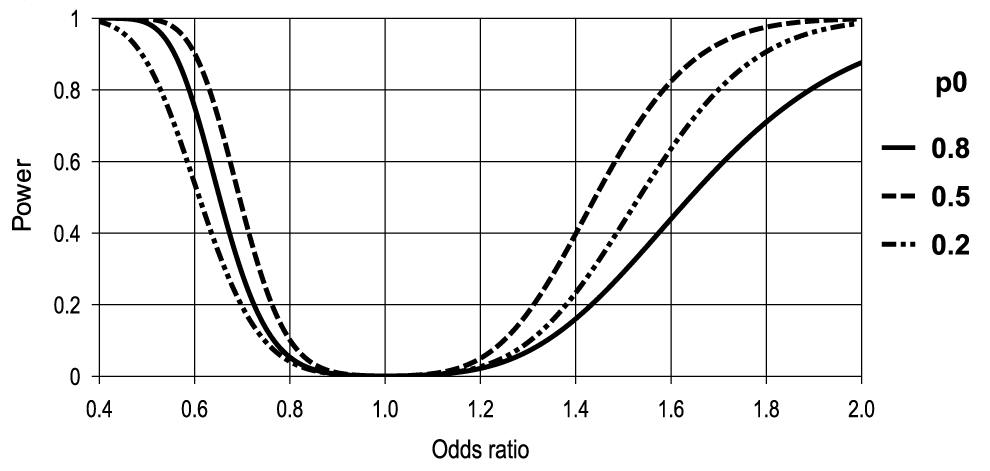

Supplement: Figure S1 — Statistical power of the AD/CRC GWAS for alleles found at different frequencies in the general population (p0). (TIF) [file pone.0035307.s003.tif]
